# Identification of *Plasmodium falciparum* VAR2CSA peptides differentially recognized by IgG of multigravidae through epitope excision

**DOI:** 10.3389/fimmu.2025.1704346

**Published:** 2025-11-10

**Authors:** Santosh A. Misal, Jonathan P. Renn, Robert D. Morrison, Matthew V. Cowles, Almahamoudou Mahamar, Oumar Attaher, Alassane Dicko, Patrick E. Duffy, Michal Fried

**Affiliations:** 1 Molecular and Pathogenesis Biomarkers Section, Laboratory of Malaria Immunology and Vaccinology (LMIV), National Institute of Allergy and Infectious Diseases (NIAID), National Institutes of Health (NIH), Bethesda, MD, United States; 2 Vaccine Development Unit, Laboratory of Malaria Immunology and Vaccinology (LMIV), National Institute of Allergy and Infectious Diseases (NIAID), National Institutes of Health (NIH), Bethesda, MD, United States; 3 Malaria Research & Training Center, Faculty of Medicine, Pharmacy and Dentistry, University of Sciences Techniques and Technologies of Bamako, Bamako, Mali; 4 Pathogenesis and Immunity Section, Laboratory of Malaria Immunology and Vaccinology (LMIV), National Institute of Allergy and Infectious Diseases (NIAID), National Institutes of Health (NIH), Bethesda, MD, United States

**Keywords:** epitope mapping, epitope excision, VAR2CSA vaccine, pregnancy malaria vaccine, *Plasmodium falciparum*, malaria

## Abstract

**Background:**

Placental malaria is associated with adverse outcomes for both mothers and their newborn children. During pregnancy, *Plasmodium falciparum*–infected erythrocytes (IEs) that surface-express VAR2CSA can bind chondroitin sulfate (CSA) in intervillous spaces and sequester in the placenta. Women acquire antibodies to VAR2CSA during their first pregnancy, but functional antibodies that block IE adhesion and are associated with improved outcomes develop over 2-3 pregnancies. Currently, VAR2CSA is the leading pregnancy malaria vaccine candidate.

**Methods:**

To identify and quantify epitopes differentially recognized by IgG of multigravidae that acquired anti-adhesion antibodies compared to primigravidae that did not, we applied epitope excision and multiplex isobaric labeling to quantify epitope recognition by naturally acquired antibodies.

**Results:**

While primigravidae and multigravidae IgG reacted similarly to most epitopes, multigravidae IgG differentially recognized (Log2 fold change > 1, p < 0.05) ten epitopes conserved across multiple VAR2CSA alleles.

**Conclusion:**

Knowledge of VAR2CSA epitopes preferentially recognized by immune multigravidae will be valuable for designing a VAR2CSA subunit vaccine.

## Introduction

Malaria during pregnancy is a major public health problem, associated with poor pregnancy outcomes such as stillbirth, preterm birth, small for gestational age and low birthweight (1–3). *Plasmodium falciparum* (Pf) infection of pregnant women is characterized by sequestration of infected erythrocytes (IEs) in the placenta, uniquely binding to placental receptor chondroitin sulfate A (CSA) expressed on the syncytiotrophoblast surface and in intervillous spaces (4). IE adhesion to CSA is mediated by VAR2CSA, a parasite surface protein and member of the *Plasmodium falciparum* erythrocyte membrane protein 1 (PfEMP1) variant antigen family (5). In high malaria transmission zones, susceptibility to pregnancy malaria is highest during the first pregnancy (6). Over successive pregnancies, women acquire antibodies to CSA-binding parasites, including anti-adhesion antibodies that reduce IE binding to CSA and associate with reduced risks of maternal infection, small for gestational age births and increased birth weight (7–11).

Naturally acquired anti-adhesion antibodies reduce placental parasite adhesion to CSA regardless of geographical origin of the parasite isolate, suggesting epitopes targeted by anti-adhesion antibodies are conserved (7). In multiple studies, antibody levels to recombinant VAR2CSA increased over successive pregnancies and were significantly higher in multigravidae compared to primigravidae (10, 12–16). Consequently, VAR2CSA is the leading candidate for a vaccine to prevent pregnancy malaria (17). However, it is unknown whether functional antibodies acquired by multigravidae target the same epitopes as those in primigravidae, or target functional epitopes not recognized by primigravidae IgG.

To dissect potential differences in naturally acquired VAR2CSA antibodies of primigravidae and multigravidae, we applied epitope mapping technology. Several methods are used to map epitopes, including peptide-library (PEPSCAN), X-ray crystallography, Hydrogen-Deuterium Exchange coupled with Mass Spectrometry (HDX-MS), Cryogenic Electron Microscopy (Cryo-EM), and epitope excision (Reviewed in (18, 19). HDX-MS is a popular method used to map epitopes based on comparing the rate of hydrogen exchange with deuterium in an antibody-antigen complex versus free antigen. The amino acid regions of the antigen are protected from deuterium exchange upon binding, and are considered as potential epitopes (20, 21). However, HDX-MS requires specialized instruments and expertise, and is often unable to differentiate between allosteric sites and true epitope binding sites (22). PEPSCAN is an old technique that recently gained renewed interest for epitope mapping, with multiple reports demonstratingits effectiveness of this approach to identify B and T-cell epitopes. However, it is confined to a focus on linear epitopes (23, 24). X-ray crystallography is also a powerful technique for high-resolution mapping of linear and conformational epitopes (25), but is limited by complex sample preparation and challenges with crystallization (26). In this study, we applied epitope excision and quantitative mass spectrometry with isobaric tandem mass tags (TMT) labeling to profile differential specificities of plasma antibody from multigravidae and primigravidae. Epitope excision followed by mass spectrometry is often used with limited proteolytic digestion of the antigen-antibody complex (19). The antibody-bound region of the antigen remains protected during proteolysis, making it a potential epitope that can be identified by mass spectrometry (19, 27). This approach has been used to identify discontinuous epitopes recognized by monoclonal antibodies (28, 29). Labeling with TMT enhances quantification precision of multiple samples analyzed together. TMT quantitative proteomics is designed to quantify the relative abundance of a peptide across multiple samples simultaneously. Multiplexing samples enables quantifying same peptides across samples, reducing missing data associated with label-free methods (30). In addition, multiplexing reduces variations between LC-MS/MS runs and provides a higher protein detection rate (31).

To generalize the finding, we compared epitopes recognized by primigravidae and multigravidae IgG across four VAR2CSA allelic forms. We identified between 199–226 epitopes across the different VAR2CSA alleles, including two epitopes identified in 4 alleles and eight epitopes identified in 3 alleles that were recognized exclusively or at significantly higher levels by multigravidae IgG.

## Methods

### Ethical approval

Plasma samples from multigravid women (MG) (n=8), and primigravid women (PG) (n=8) were collected from pregnant women enrolled into a longitudinal cohort study of mother-infant pairs carried out in Ouelessebougou, Mali (3). Samples used here were collected at delivery. The study protocol was approved by the institutional review board of the National Institutes of Health (ClinicalTrials.gov NCT01168271), and the Ethics Committee of the Faculty of Medicine, Pharmacy and Dentistry at the University of Bamako, Mali. All individual participants provided written informed consent after community permission was obtained.

### Polyclonal antibody isolation and recombinant VAR2CSA expression

IgG was purified from 50 µl plasma samples using Melon Gel IgG purification spin columns (Thermo Fisher, P/N-45206). 100 µg IgG were immobilized on 70µl Protein G Mag Sepharose Xtra (Cytiva, P/N-28967070) and incubated for 1 hour at room temperature to allow IgG binding.

Recombinant VAR2CSA alleles were prepared as previously described (16). Briefly, full-length VAR2CSA ectodomains (NTS to DBL6) were synthesized with human optimized codons. Expi293 cells were transfected with plasmid containing VAR2CSA. Culture supernatant were collected 7 days after transfection and the protein was purified on HisTrap Excel NTA column (Cytiva) and then on a size-exclusion S6 16/60 column (Cytiva). Recombinant proteins were deglycosylated using Protein Deglycosylation Mix II (New England Biolabs) according to the manufacturer’s protocol.

### Epitope excision

20 ug of recombinant VAR2CSA alleles (16) were added to immobilized IgG and incubated for 1 hour, followed by washing unbound VAR2CSA. For proteolytic digestion, 3-hour incubation with trypsin (1:20 ratio, protein-enzyme) was followed by addition of chymotrypsin (1:10 ratio, protein-enzyme) and continued digestion for an additional 3 hr. Unbound peptides were washed off the beads and bound peptides were eluted twice with 0.1% TFA. The peptides were cleaned with C18 ZipTips (Millipore Sigma), dried and stored at -80°C until further use.

### TMTpro 16plex labeling and high pH reverse phase chromatography

100 µg of each peptide sample from multigravidae and primigravidae were used for labeling with TMTpro 16 plex reagent according to the manufacturer’s protocol (Thermo Fisher Scientific). Labeled peptides were fractionated by a high pH reverse phase chromatography column (Waters, 1mm x 50mm XBridge BEH C18 column) connected to the Shimadzu HPLC. The peptide samples were loaded on a column with 10 mM Triethylammonium bicarbonate buffer (TEAB) as a mobile phase A, and solvent B was 90% Acetonitrile with 10mM TEAB over a 120-minute linear gradient.

### Liquid chromatography mass spectrometry analysis

500 ng of fractionated peptide samples were injected into a trap cartridge (0.3 mm x 5mm packed with 5 µm C18 resin, Thermo Scientific) with Buffer A, 0.1% formic acid, and Optima™ LC-MS grade water (Fisher Scientific), then separated by an Aurora Ultimate TS 25×75 C18 UHPLC column (IonOpticks, Australia) on Ultimate™ 3000 RSLC nano (Thermo Fisher Scientific) coupled to the Orbitrap Fusion™ Lumos™ Tribrid™ mass spectrometer using a gradient of 5% to 30% of solvent B (0.1% formic acid, acetonitrile) for 75 minutes, 30 to 45% for 10 min and then to 85% solvent B for additional 20 minutes. The mass spectrometer was set to scan m/z from 400 to 1800. The full MS scans were acquired using an Orbitrap at a 120,000 resolution in profile mode, followed by data-dependent MS/MS scans in the Orbitrap at 50,000 resolution with a cycle time of 3 seconds. Monoisotopic precursor selection was enabled, and charge-state filtering was enabled from 2-7. The dynamic exclusion was set to 30 seconds with an automatic gain control target of 1e6 for MS1 and 1e5 for MS2 scans.

### Data analysis

Raw data were analyzed using PEAKS online v.11 (Bioinformatics Solutions, Inc. Canada) with a custom-made proteome database of VAR2CSA and immunoglobulins. Precursor and fragment mass tolerance were set at 5 PPM and 0.02 Da, respectively. Cleavage enzymes of trypsin and chymotrypsin were selected. Fixed modifications of TMTpro16plex (N-term and K, + 304.20), carbamidomethyl (C, + 57.02), and variable modifications of oxidation (M, + 15.99) and deamidation (NQ, + 0.98) were selected. Three missed cleavage sites were allowed. The false discovery rates (FDR) for PSM and proteins were set at 1% by applying the target-decoy strategy. Proteins were quantified using the PEAKS Q module by grouping the samples into multigravid and primigravid. Normalization was set to “Auto normalization”. PEAKS auto-normalization algorithm calculates a scaling factor for each channel (label) from the total intensities of all labels to ensure that each channel has equivalent total intensity. The PEAKS Q output was exported and processed in Perseus. Significant differences in peptide epitope abundance between groups were defined as a log2 fold change of >=1 or <=-1.0 and p<0.05 by Mann Whitney-U test. Only peptides with quantifiable intensities in at least 5 of the 8 samples from one of the gravid groups were included in the analysis.

## Results

### VAR2CSA epitopes recognized at a higher level by multigravidae compared to primigravidae IgG

**Figure 1:**
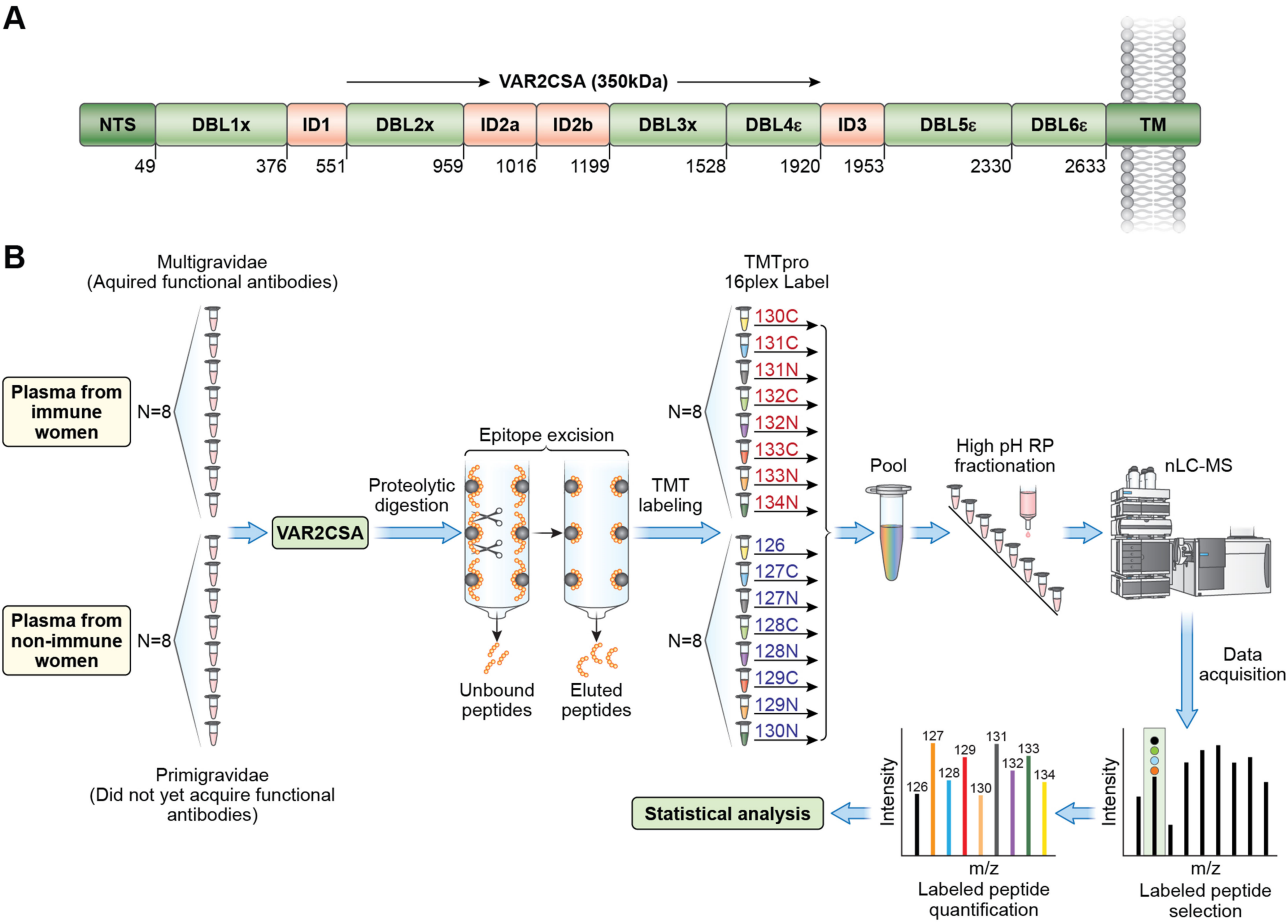
Overview of VAR2CSA protein and epitope mapping by mass spectrometry. **(A)** VAR2CSA NF54 full length protein with domain boundaries. Other VAR2CSA alleles (FCR3, HB3, and 7G8) have similar domain organization. **(B)** Summary of experimental workflow: Each VAR2CSA allele, or individual domains was incubated with plasma samples followed by digestion of unbound antigen regions and elution of bound peptides. Eluted peptides are labeled with TMT (Tamdam Mass Tag) and quantified by mass spectrometry analysis. Red font indicates multigravid samples; blue font indicates primigravid samples. .

VAR2CSA is a 350 kDa transmembrane protein. The extracellular region includes six Duffy-Binding-Like (DBL) domains and four interdomain (ID) regions (**Figure 1A**). Four allelic forms of full-length VAR2CSA (NF54, HB3, 7G8 and FCR3) (16) were used in this analysis. We employed epitope excision coupled with TMTpro labeling and mass spectrometry to identify and quantify VAR2CSA epitopes differentially recognized by IgG from multigravidae compared to primigravidae (**Figure 1B**). The analysis included plasma samples from 8 primigravidae and 8 multigravidae collected at delivery. Plasma samples included in the analysis were previously evaluated for anti-adhesion activity with heterologous fresh parasite isolates. The selected samples represented anti-adhesion activity levels observed in primigravidae and multigravidae that participated in the study (11). Anti-adhesion activity was measured with a mean (SD) of 4.8 (1.6) heterologous isolates. Median (IQR) percent inhibition was significantly higher in multigravidae [81.16 (78.49-86.70)] than primigravidae [3.46 (-6.16-31.06); p=0.0008].

A total of 225, 175, 199 and 206 peptides were quantified in NF54, HB3, 7G8 and FCR3 VAR2CSA alleles, respectively (**Supplementary Table S1**, **Supplementary Data File 1**). Two epitopes were recognized almost exclusively by multigravidae IgG across the 4 VAR2CSA variants: one mapped to DBL2 and the other to DBL4 (**Table 1**). Intensities of 8 epitopes were significantly higher with multigravidae IgG across 3 allelic forms, and these mapped to VAR2CSA domains DBL1, ID1, DBL2, ID2b, DBL3, and DBL4 (**Table 1**). Five of the eight epitopes were identified in the fourth allele as well, however neither fold change nor p value met the criteria for differentially recognized epitopes (log_2_ fold change <=-1.0 or >=1.0, p<0.05) (**Table 1**). In addition, intensities of 14 epitopes were significantly higher with multigravidae IgG in two VAR2CSA allelic forms (**Supplementary Table S2**). We also identified allele-specific epitopes differentially recognized by multigravidae IgG (**Supplementary Table S3**).

**Table 1:** Differentially recognized epitopes across 3-4 alleles.

Full-length VAR2CSA								Domain analysis			
Allele	Epitope	Start-end	Log2 FC	P-value	Domain	PG (n)	MG (n)	Log2 FC	P-value	PG (n)	MG (n)
NF54	DLELNLQK	670-677	8.301	0.01	DBL2	0	5	7.43	0.01	0	5
HB3	DLELNLQK	672-679	10.20	0.01		0	5				
FCR3	NLQNNFGK	676-683	16.42	0.002		1	8				
7G8	DLELNLQK	676-683	5.34	0.01		0	5				
NF54	ELFPIIIK	1617-1624	12.07	0.01	DBL4	0	5	6.03	0.01	0	5
HB3	ELFPIIIK	1649-1656	12.36	0.01		0	5				
FCR3	ELFPIIIK	1635-1642	12.7	0.01		0	5				
7G8	ELFPIIIK	1645-1652	6.83	0.01		0	5				
NF54	GVQHIGIAK	1779-1787	8.78	0.02	DBL4	3	8				
FCR3	GVEHIGIAK	1784-1792	11.49	0.04		3	8				
HB3	GVEHIGIAK	1806-1814	8.99	0.008		3	8				
7G8	^1^IGIAKPQ	1804-1810	0.3	0.016		8	8				
NF54	SNDLLIKR	231-238	16.46	0.001	DBL1	0	7				
FCR3	SNDLLIKR	232-239	15.98	0.001		0	7				
7G8	SNDLLIKR	235-242	5.01	0.001		0	8				
HB3	^1^SNDLLIK	232-238	0.18	0.06		8	8				
NF54	SEWENQK	337-343	11.72	0.004	DBL1	3	8				
HB3	SEWENQK	338-344	12.29	0.001		3	8				
7G8	SEWENQK	341-347	6.21	0.008		3	8				
FCR3	^1^ENQENK	341-346	-0.15	0.87		8	8				
NF54	SSLENYIK	381-388	14.64	0.001	ID1	0	7	10.30	0.001	0	7
FCR3	SSLENYIK	382-389	14.89	0.001		0	7				
7G8	SSLENYIK	387-394	5.62	0.001		0	7				
HB3	^1^SSANSY	382-387	-0.03	0.564		8	8				
NF54	LGVRENDK	467-474	14.13	0.001	ID1	1	8	6.21	0.0005	1	8
FCR3	LGVRENDK	468-475	13.15	0.009		1	8				
7G8	LGVRENDK	473-480	5.53	0.002		1	8				
HB3	^1^LGINNNDK	468-475	0.33	0.318		7	8				
NF54	FLQEWVEHF	760-768	12.67	0.002	DBL2	1	8	7.71	0.003	1	8
HB3	FLQEWVEHF	762-770	15.24	0.001		1	8				
FCR3	FLQEWVENF	762-770	4.24	0.027		5	8				
NF54	ENESTNNK	1195-1202	15.94	0.002	ID2b	1	8				
7G8	ENESTDTK	1201-1208	1.60	0.009		3	8				
HB3	EIQNTDTK	1207-1214	14.52	0.001		0	7				
NF54	NMILGTSVNIY	1351-1361	10.75	0.003	DBL3	3	8				
FCR3	NMILGTSVNIY	1356-1366	13.03	0.003		3	8				
HB3	NMILGTSVNIY	1372-1382	11.26	0.001		3	8				
7G8	NMILGTSVNTY	1366-1376	2.84	0.1		3	8				

^1^Peptide did not meet cutoff criteria of fold change (log2 fold <=-1.0 or >=1) or p value <0.05.

Intensities of 2, 7, 3 and 14 epitopes in NF54, HB3, 7G8 and FCR3 alleles, respectively, were significantly higher with primigravidae IgG. However, log2 fold change did not meet the cutoff criteria except for one peptide epitope in HB3 allelic form (amino acids 2600-2605, **Supplementary Table S4).**

The conservation of amino acid residues across 765 VAR2CSA variant forms was evaluated for the peptide epitopes identified by our method. The median (IQR) of amino acid conservation in those identified in one, two, three or four alleles were respectively 89.29 (61.52-100), 91.61 (63.16-100), 99.41 (76.35-100) and 100 (86.71-100). Amino acid conservation levels differed significantly between those identified in 3–4 versus 1–2 alleles (adjusted p<0.01), as well as in 4 versus 3 alleles (adjusted p<0.01). Sequence conservation of the ten peptide epitopes preferentially recognized by multigravidae IgG in 3–4 alleles ranged between 71.66-99.87% (**Supplementary Table S5**).

### Comparison of epitopes differentially recognized in full-length VAR2CSA and VAR2CSA domains

We then evaluated whether differentially recognized epitopes across 3–4 allelic forms are also differentially recognized by multigravidae IgG when presented as part of single or double domains. For this experiment, we developed two recombinant proteins, ID1-DBL2-ID2a fragment and DBL4 domain (amino acids L376-D1016 and I1529-L1920, respectively) based on the NF54 allele sequence. Recombinant ID1-DBL2-ID2a contains the entire sequence of one of the first two vaccines tested in human vaccine trials, named PAMVAC (32). The same plasma samples were used in this analysis as in the analysis of full-length VAR2CSA, and we followed the same procedure described above. Fifty-seven and 32 peptide epitopes in ID1-DBL2-ID2a and DBL4 recombinant domains, respectively, were quantified, of which 39/57 and 26/32 were shared with peptides identified in the analysis of full-length VAR2CSA (**Supplementary Data File 1**). Three peptides from ID1-DBL2-ID2a fragment and 12 from DBL4 domain were identified in the analysis of full-length VAR2CSA but not the domains analysis (**Supplementary Data File 1**). The 4 epitopes in ID1-DBL2 domains, and 1 of the 2 epitopes in DBL4, identified across 3–4 allelic forms in the analysis of full-length VAR2CSA (described above), were also identified in the analysis of ID1-DBL1-ID2a (**Table 2**) as differentially reactive to multigravidae; the second DBL4 peptide (amino acids G1779-K1787 in NF54 allele) was not identified in the domain analysis, suggesting DBL4 alone did not recapitulate the epitope structure (**Table 2**).

**Table 2:** Differentially recognized epitopes identified in ID1-ID2a and DBL4 domains.

Domain	Epitope	Start-end	Log2 FC	P value	Domain	PG (n)	MG (n)
ID1-ID2a (NF54)	^1^SSLENYIK	381-388	10.30	0.001	ID1	0	7
	^1^LGVRENDK	467-474	6.21	0.0005	ID1	1	8
	^1^NNKNWIW	554-560	3.76	0.01	DBL2	0	5
	^2^EGGLQKEY	567-574	6.79	0.02	DBL2	3	8
	^1^EYANTIGLPPR	573-583	5.29	0.02	DBL2	3	8
	^1^DLELNLQK	670-677	7.43	0.01	DBL2	0	5
	^2^SSLDELRESW	700-709	3.78	0.03	DBL2	3	8
	^3^YIWLAMK	716-722	7.90	0.0005	DBL2	1	8
	^1^FLQEWVEHF	760-768	7.71	0.003	DBL2	1	8
	^1^TTYTTTEK	912-919	7.28	0.0006	DBL2	1	8
DBL4 (NF54)	^1^NDNIEYK	1538-1544	2.62	0.03	DBL4	5	8
	^3^YKTYYP	1543-1548	1.10	0.0008	DBL4	8	8
	^3^KTYYPY	1544-1549	1.09	0.0008	DBL4	8	8
	^2^GVYVPPRR	1603-1610	1.19	0.046	DBL4	8	7
	^1^ELFPIIIK	1617-1624	6.03	0.01	DBL4	0	5
	^1^QYHAHNDTTY	1654-1663	4.38	0.001	DBL4	1	8
	^2^SDPKTIR	1740-1746	1.20	0.003	DBL4	8	8
	^2^VWDAMQSGVR	1749-1758	1.07	0.002	DBL4	8	8
	^1^WLEEWTNEF	1793-1801	2.21	0.001	DBL4	0	7
	^2^ESEDGKDY	1868-1875	5.28	0.002	DBL4	1	8

^1^Similar result to that observed with full-length VAR2CSA, NF54 allele.

^2^Peptide identified in full-length VAR2CSA, but differences between primigravidae and multigravidae were not significant.

^3^Peptide was not identified in the analysis of full-length VAR2CSA.

### Structural analysis of epitopes

**Figure 2:**
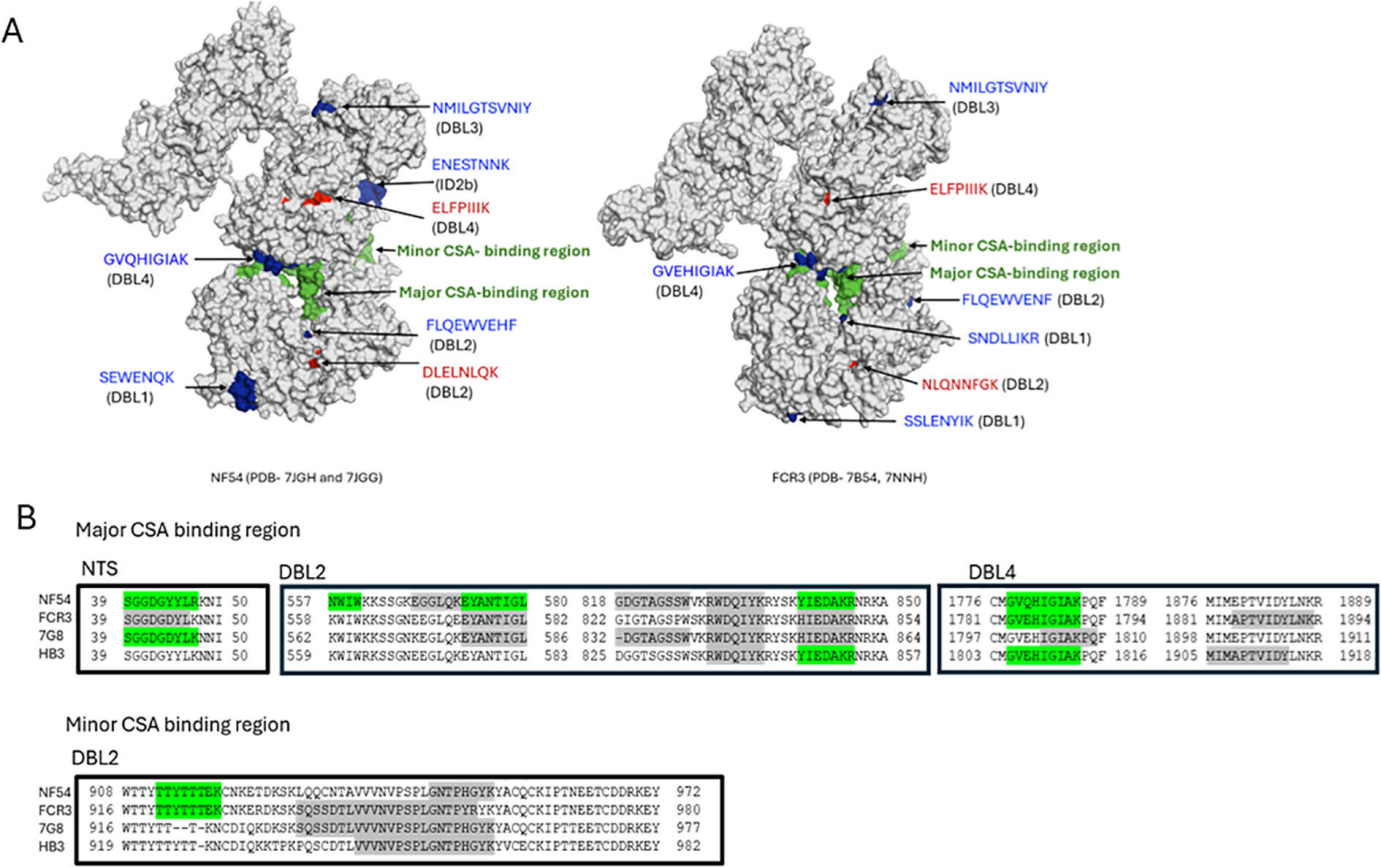
Localizing epitopes on VAR2CSA structure and in CSA binding channel. **(A)** The spatial distribution of the epitopes on 3D structures of VAR2CSA FCR3 and NF54 alleles was visualized using PyMOL software. NF54 and FCR3 structures are shown with major and minor CSA binding channels in green. Epitopes differentially recognized by multigravidae IgG across 4 alleles (in red) and across 3 alleles (in blue) are indicated. Epitope LGVRENKD in ID1 mapped to an unresolved region in both alleles, while epitope ENESTNNK localized in NF54 but not FCR3, and epitope SSLENYIK did not localize in the NF54 3D structure due to unresolved residues. **(B)** Peptides observed in major and minor CSA-binding channels of VAR2CSA. Differentially recognized peptides by multigravidae are highlighted green and peptides recognized similarly by primigravidae and multigravidae are highlighted gray. Binding channel boundaries defined according to Ma et al. analysis (34).

Epitopes differentially recognized by multigravidae IgG in the full-length VAR2CSA were displayed on 3D structures of VAR2CSA NF54 and FCR3 using PyMOL software (Schrödinger, Inc.). VAR2CSA-FCR3 and VAR2CSA-NF54 3D Cryo-EM structures were downloaded from Protein Data Bank (VAR2CSA FCR3 NTS-DBL4 and DBL5-DBL6, PDB IDs- 7B54, 7NNH; VAR2CSA-NF54 NTS-DBL4 and DBL5-DBL6, PDB IDs- 7JGH, 7JGG). The core structure of VAR2CSA encompassing NTS-DBL4 domains, and the flexible arm encompassing domains DBL5-DBL6, were determined separately for both allelic forms (33, 34). The core structure contains the major and minor CSA binding channel (34). The major CSA-binding channel includes 2 discontinued binding sites (34). To present a comprehensive view of the full-length VAR2CSA structure, the two parts of the structures were combined in PyMOL (**Figure 2A**).

The ten epitopes differentially recognized by multigravidae IgG across 3–4 alleles are located in the core structure. Of those, seven epitopes were localized to structures of one or both variants; three epitopes mapped to unresolved regions and hence were not localized (**Figure 2A**).

Based on this analysis, seven epitopes localize to the surface of VAR2CSA. One epitope in DBL4 (VAR2CSA-NF54 amino acids 1779-1787) exclusively recognized by multigravidae IgG localized in the second site of the major binding channel. Additional peptide epitopes that localized within the major or minor CSA-binding channel were identified and quantified, but were either differentially recognized by multigravidae IgG in only 1–2 alleles or intensities were similar between multigravidae and primigravidae IgG (**Figure 2B**).

## Discussion

VAR2CSA is the leading target for a pregnancy malaria vaccine but its development has been hindered by antigen size and sequence variation. The first two VAR2CSA-based vaccine candidates tested in humans, PRIMVAC (domains DBL1-ID1-DBL2) and PAMVAC (domains ID1-DBL2-ID2a), were immunogenic but elicited only allele-specific functional antibodies (32, 35). Unlike single-domain VAR2CSA recombinant proteins, full-length recombinant VAR2CSA depletes strain-transcending functional antibodies from multigravidae IgG, suggesting that individual domains may not recapitulate conformational epitopes (36, 37), or that a combination of antibodies targeting epitopes mapped to additional domains are required. In this study, we used naturally acquired polyclonal antibodies from pregnant women to compare epitope recognition between multigravidae and primigravidae IgG. We report that intensities of most epitopes were similar when IgG samples from primigravidae or multigravidae were used (**Supplementary Table S1**), consistent with the observation in this population, that during the first pregnancy, women acquire antibodies to VAR2CSA domains (15). We identified two conserved epitopes (from DBL2 and DBL4 domains) that bind exclusively or at significantly higher levels to multigravidae IgG across four VAR2CSA alleles, and eight such epitopes across three alleles (**Table 1**). Alignment of 765 VAR2CSA sequences showed a high degree of sequence conservation of these 10 peptide epitopes (**Supplementary Table S5**).

Previous analysis of IgG reactivity with VAR2CSA peptides described that peptides in DBL3 and DBL5 domains corresponding to amino acids 1350–1370 and 2045–2061 in 3D7 respectively are highly conserved, exposed on the surface of VAR2CSA and therefore accessible to antibodies. These peptides were preferentially recognized by plasma from females compared to males (38). In the current study, the peptide from DBL3 preferentially recognized by multigravidae IgG in 3 allelic forms is within the described region (**Table 1**). We also identified a peptide in DBL5 domain within the described region that was differentially recognized by multigravidae in 2 VAR2CSA alleles while there were no differences between primigravidae and multigravidae in the other 2 alleles (**Supplementary Table S2** and **Supplementary Data File 1**). Previously, a peptide array of DBL4 domain identified a peptide preferentially recognized by sera from women that acquired anti-adhesion antibodies compared to women without anti-adhesion activity (39). This peptide was also identified in our study; however, the peptide was differentially recognized by IgG of multigravidae that acquired functional anti-adhesion antibodies compared to primigravidae that did not acquire functional antibodies in NF54 allele, while intensities were similar between primigravidae and multigravidae in the other 3 alleles (**Supplementary Table S3**).

Cryo-EM studies have suggested the protein core is made of four DBL domains and 3 interdomain (ID) regions (NTS-ID3) that form a compact structure with the DBL4 domain at the center and multiple inter-domain interactions (33, 34). CSA-binding sites are within two channels in the protein core, defined as a major binding channel containing two discontinuous binding sites and a minor binding channel (34). The two epitopes (in DBL2 and DBL4) differentially recognized across the 4 alleles are outside the CSA binding channel. Of the eight epitopes differentially recognized across 3 alleles, one epitope in DBL4 domain localized to the second binding site of the major binding channel between amino acids 1779–1787 of the NF54 allele (**Table 1**, peptide GVQHIGIAK) and contains one CSA-binding residue. This peptide was not identified in single-domain DBL4 analyses, suggesting that epitope conformation may have been lost. Alternatively, this epitope could be part of a discontinuous epitope involving sequences in other domains or inter-domain regions. Additional peptides mapped to the major and minor CSA-binding channels, however these epitopes were either differentially recognized by multigravidae IgG in only 1–2 alleles or were similarly recognized by IgG of primigravidae and multigravidae (**Figure 2B**). These results suggest that by the end of the first pregnancy, women have acquired antibodies to multiple epitopes located in CSA-binding channels, and that epitopes outside CSA-binding channels may be important targets for strain-transcending functional antibodies.

Earlier adhesion studies using recombinant VAR2CSA fragments concluded that ID1-DBL2-ID2a contains the minimal CSA-binding region (17). While the first two VAR2CSA vaccines encompassed the minimal CSA binding domain, these candidates induced homologous functional activity (ie, only inhibited adhesion of IEs expressing the VAR2CSA variant in the vaccine) (32, 35). Here, four epitopes that mapped to ID1 and DBL2 regions were differentially recognized by multigravidae IgG in both the minimal CSA-binding region and full-length VAR2CSA, suggesting their conformations were similar (**Table 1**). We speculate that the combination of antibodies targeting multiple epitopes including those outside the minimal CSA-binding region (like DBL3 and DBL4 domains) are important for total serum functional activity, in which case a vaccine based on full-length VAR2CSA, or at least on the core protein (NTS-DBL4), may be required to induce antibodies with broad anti-adhesion activity to heterologous parasites.

Previous mass spectrometry analysis of inter- and intra-domain interactions described disulfide bonds and crosslinking of proximal lysine residues that play a role in stabilizing the compact VAR2CSA structure (40). Among the ten peptide epitopes differentially recognized across 3–4 alleles, three lysine-containing epitopes are involved in inter-domain interactions and two in intra-domain interactions, including an ID1 peptide (L467-K474) that interacts with several lysine residues in DBL4. This suggests acquisition of antibodies targeting inter-domain interactions may be important for developing protective immunity to placental parasites. Future studies may compare anti-adhesion activity by antibodies induced with mutated lysine residues involved in inter- and intra-domain interactions versus native protein.

In this study, we mapped and quantified VAR2CSA epitopes that are recognized exclusively or at significantly higher levels by multigravidae IgG compared to primigravidae IgG. Of the ten differentially recognized epitopes, only one localized to the CSA-binding channel; additional epitopes in the CSA-binding channels were differentially recognized by multigravidae IgG in 1–2 alleles or similarly by primigravidae and multigravidae IgG. These results highlight epitopes outside the CSA-binding region that preferentially react to multigravidae antibodies and thus may contribute to protective immunity. Further studies are needed to evaluate the contribution of these peptide epitopes to acquisition of functional anti-adhesion antibodies. For example, multigravidae IgG purified on VAR2CSA containing mutations at the epitope sites could be compared to IgG purified on native VAR2CSA for anti-adhesion activity.

### Limitations of the study

A limitation of our study is that we cannot determine whether any of the peptide epitopes are part of discontinuous epitopes. Unlike earlier studies that mapped discontinuous epitopes by epitope excision method with monoclonal antibodies, here we used human polyclonal IgG that reacts with numerous epitopes. Hence, after eluting IgG-bound fragments from immobilized IgG, it was not possible to determine whether any of the peptides are part of a discontinuous epitope.

## Data Availability

The original contributions presented in the study are included in the article/**Supplementary Material**. Further inquiries can be directed to the corresponding author.
