# Supplementary material for: Identification of Plasmodium falciparum VAR2CSA peptides differentially recognized by IgG of multigravidae through epitope excision
Source: Front Immunol. 2025 Nov 10;16:1704346. doi: 10.3389/fimmu.2025.1704346 (PMC12641020; doi:10.3389/fimmu.2025.1704346)
Supplement: Supplementary file 2 [file Table1.docx]

**Identifying epitopes on *Plasmodium falciparum* VAR2CSA differentially recognized by multigravidae IgG by epitope excision**

Santosh A. Misal, Jonathan P. Renn, Robert D. Morrison, Matthew V. Cowles, Alassane Dicko, Patrick E. Duffy, Michal Fried

**SUPPLEMENTARY MATERIAL**

Supplementary Tables S1-S5

**Supplementary Table S1. Total identified and quantified peptides in each VAR2CSA allele**

| **Allele** | **Total peptides (MG+PG)** | **DR peptides in MG** | **DR peptides >2 alleles in MG** | **DR in MG allele Specific peptides** | **DR peptides in PG** |
| --- | --- | --- | --- | --- | --- |
| VAR2CSA_NF54 | 226 | 53 | 21 | 32 | 0 |
| VAR2CSA_HB3 | 175 | 21 | 12 | 9 | 0 |
| VAR2CSA_FCR3 | 206 | 20 | 13 | 7 | 0 |
| VAR2CSA_7G8 | 199 | 18 | 15 | 3 | 1 |

Total VAR2CSA peptides quantified in multigravidae (MG) and primigravidae (PG) plasma samples. No. of differentially recognized (DR) (Log2 FC >1, p-value <0.05) peptides in MG and DR allele specific peptides

**Supplementary Table S2. Differentially recognized epitopes identified in two alleles**

| **Protein** | **Epitope** | **Start-end** | **Log2 FC** | **P-value** | **Domain** | **PG (n)** | **MG (n)** |
| --- | --- | --- | --- | --- | --- | --- | --- |
| VAR2CSA_7G8 | SGGDGDYLK | 39-47 | 3.9 | 0.002 | NTS | 5 | 8 |
| VAR2CSA_NF54 | GSGGDGYYLR | 38-47 | 2.57 | 0.04 |  | 7 | 8 |
|  |  |  |  |  |  |  |  |
| VAR2CSA_FCR3 | LPPYDDNDQW | 71-80 | 10.02 | 0.001 | DBL1 | 0 | 8 |
| VAR2CSA_HB3 | ELSYGDNDQW | 71-80 | 7.77 | 0.040 |  | 3 | 8 |
|  |  |  |  |  |  |  |  |
| VAR2CSA_NF54 | TTYTTTEK | 912-919 | 14.61 | 0.001 | DBL2 | 1 | 8 |
| VAR2CSA_FCR3 | TTYTTTEK | 921-928 | 13.43 | 0.003 |  | 1 | 8 |
|  |  |  |  |  |  |  |  |
| VAR2CSA_NF54 | GSQNKK | 1123-1128 | 9.62 | 0.010 | ID2b | 0 | 7 |
| VAR2CSA_FCR3 | GSQNKK | 1132-1137 | 11.71 | 0.010 |  | 0 | 7 |
|  |  |  |  |  |  |  |  |
| VAR2CSA_7G8 | EAYYLWK | 1675-1681 | 1.00 | 0.010 | DBL4 | 0 | 7 |
| VAR2CSA_HB3 | EAYYLWK | 1679-1685 | 9.29 | 0.010 |  | 0 | 7 |
|  |  |  |  |  |  |  |  |
| VAR2CSA_NF54 | ESEDGKDY | 1868-1875 | 13.11 | 0.002 | DBL4 | 1 | 8 |
| VAR2CSA_7G8 | ESEDGKDY | 1889-1896 | 5.39 | 0.001 |  | 1 | 8 |
|  |  |  |  |  |  |  |  |
| VAR2CSA_NF54 | KLYPLDR | 1978-1984 | 6.83 | 0.002 | DBL5 | 5 | 8 |
| VAR2CSA_HB3 | KLYPLDR | 2007-2013 | 6.17 | 0.002 |  | 5 | 8 |
|  |  |  |  |  |  |  |  |
| VAR2CSA_NF54 | KGVLIPPR | 2029-2036 | 2.67 | 0.01 | DBL5 | 7 | 8 |
| VAR2CSA_FCR3 | GVLIPPRRR | 2036-2044 | 1.06 | 0.01 |  | 8 | 8 |
|  |  |  |  |  |  |  |  |
| VAR2CSA_FCR3 | IVRGPANLR | 2051-2059 | 1.32 | 0.009 | DBL5 | 8 | 8 |
| VAR2CSA_HB3 | IVRGPANLR | 2074-2082 | 1.00 | 0.006 |  | 8 | 8 |
|  |  |  |  |  |  |  |  |
| VAR2CSA_NF54 | DYEYIIK | 2096-2102 | 12.82 | 0.002 | DBL5 | 1 | 8 |
| VAR2CSA_7G8 | DYEDIIK | 2125-2131 | 3.67 | 0.009 |  | 5 | 8 |
|  |  |  |  |  |  |  |  |
| VAR2CSA_NF54 | SIQWEAISEGY | 2233-2243 | 9.18 | 0.010 | DBL6 | 0 | 5 |
| VAR2CSA_7G8 | SIQWEAISER | 2265-2274 | 3.19 | 0.014 |  | 3 | 8 |
|  |  |  |  |  |  |  |  |
| VAR2CSA_NF54 | EAISEGYK | 2237-2244 | 6.74 | 0.009 | DBL6 | 5 | 8 |
| VAR2CSA_7G8 | EAISERY | 2269-2275 | 1.08 | 0.002 |  | 8 | 8 |
|  |  |  |  |  |  |  |  |
| VAR2CSA_NF54 | GNDYICNK | 2329-2336 | 9.76 | 0.03 | DBL6 | 3 | 8 |
| VAR2CSA_FCR3 | GNDYICNK | 2326-2333 | 9.80 | 0.03 |  | 3 | 8 |
|  |  |  |  |  |  |  |  |
| VAR2CSA_7G8 | ATLEDTFK | 2645-2652 | 5.96 | 0.006 | DBL6 | 3 | 8 |
| VAR2CSA_HB3 | ETLEETLK | 2647-2654 | 15.80 | 0.001 |  | 0 | 7 |

**Supplementary Table S3. Differentially recognized epitopes identified in individual alleles**

| **Protein** | **Epitope** | **Start-end** | **Log2 FC** | **P-value** | **Domain** | **PG (n)** | **MG (n)** |
| --- | --- | --- | --- | --- | --- | --- | --- |
| VAR2CSA_7G8 | NEIDNAIK | 1990-1997 | 2.44 | 0.020 | DBL5 | 3 | 8 |
|  | NAEDWWK | 2162-2168 | 6.93 | 0.001 | DBL5 | 0 | 8 |
|  | SLQASESTK | 2241-2249 | 7.80 | 0.001 | DBL6 | 0 | 8 |
| VAR2CSA_FCR3 | DNNAFLADVLL | 120-130 | 4.58 | 0.027 | DBL1 | 5 | 8 |
|  | GKLFGK | 682-687 | 12.46 | 0.001 | DBl2 | 0 | 7 |
|  | SLEGVYVPPRR | 1618-1628 | 9.05 | 0.010 | DBL4 | 0 | 5 |
|  | LDELDEWNDM | 2016-2025 | 7.66 | 0.010 | DBL5 | 0 | 5 |
|  | NNNEDNEK | 2288-2295 | 8.52 | 0.010 | DBL5 | 0 | 5 |
|  | NYILTK | 2554-2559 | 1.13 | 0.046 | DBL6 | 8 | 8 |
| VAR2CSA_HB3 | DSTSTIGDK | 2-10 | 9.171 | 0.020 | NTS | 3 | 8 |
|  | SLSGVDNCCCQD | 486-497 | 8.514 | 0.009 | ID1 | 1 | 8 |
|  | TYNGVQIK | 1009-1016 | 10.69 | 0.001 | ID2a | 3 | 8 |
|  | KIIEQER | 1394-1400 | 11.791 | 0.025 | DBL3 | 3 | 8 |
|  | AIDEENEK | 1787-1794 | 15.35 | 0.001 | DBL4 | 0 | 7 |
|  | SMIMAPTVIDY | 1903-1913 | 9.59 | 0.005 | DBL4 | 0 | 7 |
|  | KYQELIR | 2262-2268 | 14.956 | 0.001 | DBL5 | 0 | 7 |
|  | NNDNIW | 2375-2380 | 6.132 | 0.030 | DBL6 | 3 | 8 |
|  | YIEQIFK | 2477-2483 | 15.498 | 0.001 | DBL6 | 1 | 8 |
| VAR2CSA_NF54 | SNLEQNLK | 176-183 | 12.812 | 0.001 | DBL1 | 1 | 8 |
|  | DQNYRK | 203-208 | 1.00 | 0.020 | DBL1 | 8 | 8 |
|  | IKGDPY | 387-392 | 2.128 | 0.001 | ID1 | 8 | 8 |
|  | NNKNWIW | 554-560 | 9.526 | 0.010 | DBL2 | 0 | 5 |
|  | EYANTIGLPPR | 573-583 | 10.66 | 0.002 | DBL2 | 3 | 8 |
|  | NDDNGK | 635-640 | 1.049 | 0.006 | DBL2 | 8 | 8 |
|  | YIEDAK | 840-845 | 1.0 | 0.020 | DBL2 | 8 | 8 |
|  | YIEDAKR | 840-846 | 3.45 | 0.010 | DBL2 | 7 | 8 |
|  | GYKNDNY | 987-993 | 1.30 | 0.036 | ID2a | 8 | 8 |
|  | VSDEAAQPK | 1058-1066 | 17.47 | 0.001 | ID2b | 1 | 8 |
|  | FSDNERDR | 1067-1074 | 1.15 | 0.046 | ID2b | 8 | 8 |
|  | NSITHEDK | 1075-1082 | 17.01 | 0.002 | ID2b | 1 | 8 |
|  | SITHEDK | 1076-1082 | 1.18 | 0.002 | ID2b | 8 | 8 |
|  | INDQWDK | 1098-1104 | 11.28 | 0.010 | ID2b | 3 | 8 |
|  | LYEYHDK | 1302-1308 | 9.90 | 0.010 | DBL3 | 0 | 5 |
|  | NNDSNGLPK | 1328-1336 | 14.37 | 0.001 | DBL3 | 0 | 7 |
|  | GIEGEMWDAVR | 1400-1410 | 9.25 | 0.001 | DBL3 | 0 | 7 |
|  | NDNIEYK | 1538-1544 | 5.9 | 0.002 | DBL3 | 5 | 8 |
|  | QYHAHNDTTY | 1654-1663 | 11.214 | 0.001 | DBL4 | 1 | 8 |
|  | ENEAIAVPN | 1725-1733 | 6.108 | 0.030 | DBL4 | 1 | 6 |
|  | WLEEWTNEF | 1793-1801 | 9.678 | 0.001 | DBL4 | 0 | 7 |
|  | RIEWNGMSNY | 1848-1857 | 9.081 | 0.010 | DBL4 | 0 | 5 |
|  | IEWNGMSNYY | 1849-1858 | 7.789 | 0.01 | DBL4 | 0 | 5 |
|  | ETQCEDNK | 1924-1931 | 8.435 | 0.010 | ID3 | 0 | 5 |
|  | NDVDMR | 2017-2022 | 1.30 | 0.027 | DBL5 | 8 | 8 |
|  | NDVDMRDPY | 2017-2025 | 1.04 | 0.036 | DBL5 | 8 | 8 |
|  | NDMQEITK | 2285-2292 | 10.712 | 0.010 | DBL5 | 0 | 7 |
|  | EQVDIPAEL | 2306-2314 | 2.931 | 0.006 | DBL5 | 7 | 8 |
|  | GEAKTK | 2414-2419 | 13.648 | 0.001 | DBL6 | 1 | 8 |
|  | ESPEYFK | 2589-2595 | 7.434 | 0.009 | DBL6 | 5 | 8 |
|  | ESPEYF | 2589-2594 | 1.00 | 0.020 | DBL6 | 8 | 8 |
|  |  |  |  |  |  |  |  |

**Supplementary Table S4. Peptides differentially recognized by primigravid IgG.**

| **Protein** | **Peptide** | **Start-end** | **Log2 FC** | **P-value** |
| --- | --- | --- | --- | --- |
| **2 alleles** |  |  |  |  |
| VAR2CSA_NF54 | GDPYFAEY | 389-396 | -0.68 | 0.006 |
| VAR2CSA_FCR3 | GDPYFAEY | 390-397 | -0.61 | 0.006 |
| VAR2CSA_FCR3 | YHIWESML | 2470-2477 | -0.51 | 0.04 |
| VAR2CSA_7G8 | YHIWESML | 2499-2506 | -0.33 | 0.046 |
|  |  |  |  |  |
| **Allele specific** |  |  |  |  |
| VAR2CSA_NF54 | YTNIGNEAF | 2293-2301 | -0.51 | 0.02 |
| VAR2CSA_HB3 | HLIDIGL | 891-897 | -0.40 | 0.006 |
|  | AAGTLK | 1018-1023 | -0.33 | 0.01 |
|  | IYEYIGK | 1381-1387 | -0.22 | 0.01 |
|  | CTINGK | 1490-1495 | -0.42 | 0.02 |
|  | GSSNKN | 1739-1744 | -0.44 | 0.03 |
|  | IEQIFK | 2478-2483 | -0.30 | 0.03 |
|  | SHYDMN | 2600-2605 | -1.88 | 0.04 |
| VAR2CSA_FCR3 | ADPSEVEYY | 30-38 | -0.41 | 0.02 |
|  | IRENDK | 190-195 | -0.31 | 0.046 |
|  | WDQIYK | 835-840 | -0.28 | 0.046 |
|  | HIEDAKR | 845-851 | -0.43 | 0.03 |
|  | LDGNDVTFF | 1024-1032 | -0.40 | 0.02 |
|  | DNYNKF | 1116-1121 | -0.55 | 0.006 |
|  | GTPQQK | 1383-1388 | -0.47 | 0.03 |
|  | IGGVGSSTENVN | 1391-1402 | -0.43 | 0.009 |
|  | DAMQSGVR | 1762-1769 | -0.58 | 0.04 |
|  | VIDYLNK | 1888-1894 | -0.49 | 0.006 |
|  | GTDMLTNIEF | 2109-2118 | -0.30 | 0.046 |
|  | IPAELEDVIY | 2307-2316 | -0.45 | 0.02 |
| VAR2CSA_7G8 | QIVAEREAY | 1669-1677 | -0.65 | 0.002 |
|  | NLFLNIK | 2401-2407 | -0.79 | 0.001 |

**Supplementary Table S5. Percent conserved amino acids across 765 VAR2CSA isolates**

| **Peptide (Domain)** | **Conservation** | **Consensus AA (%)** | **Mean conservation (%)** |
| --- | --- | --- | --- |
| **DLELNLQK** (DBL2) |  |  | 95.78 |
| **D** | 93.8 | D:93.8; N:6.2 |  |
| **L** | 93.7 | L:93.7; V:6.1; M:0.3 |  |
| **E** | 100 | E:100 |  |
| **L** | 100 | L:100 |  |
| **N** | 100 | N:100 |  |
| **L** | 100 | L:100 |  |
| **Q** | 100 | Q:100 |  |
| **K** | 78.8 | K:78.8; Q:15; N:6.2 |  |
| **ELFPIIIK** (DBL4) |  |  | 99.87 |
| **E** | 100 | E:100 |  |
| **L** | 99.1 | L:99.1; I:0.9 |  |
| **F** | 99.9 | F:99.9; Y:0.1 |  |
| **P** | 100 | P:100 |  |
| **I** | 100 | I:100 |  |
| **I** | 100 | I:100 |  |
| **I** | 100 | I:100 |  |
| **K** | 100 | K:100 |  |
| **GVQHIGIAK** (DBL4) |  |  | 95.54 |
| **G** | 73.9 | G:73.9; R:25.6; E:0.4; S:0.1 | |
| **V** | 99.4 | V:99.4; I:0.6 |  |
| **E** | 87.6 | E:87.6; Q:12.4 |  |
| **H** | 99.9 | H:99.9; R:0.1 |  |
| **I** | 100 | I:100 |  |
| **G** | 100 | G:100 |  |
| **I** | 100 | I:100 |  |
| **A** | 99.2 | A:99.2; G:0.8 |  |
| **K** | 99.9 | K:99.9; N:0.1 |  |
| **P** | 100 | P:100 |  |
| **Q** | 100 | Q:100 |  |
| **SNDLLIKR** (DBL1) |  |  | 99.87 |
| **S** | 100 | S:100 |  |
| **N** | 100 | N:100 |  |
| **D** | 100 | D:100 |  |
| **L** | 100 | L:100 |  |
| **L** | 99.9 | L:99.9; F:0.1 |  |
| **I** | 100 | I:100 |  |
| **K** | 100 | K:100 |  |
| **R** | 99.1 | R:99.1; L:0.9 |  |
| **SEWENQK** (DBL1) |  |  | 87.62 |
| **S** | 47.9 | S:47.9; T:41.7; K:8.4; N:2.1 | |
| **E** | 97.9 | E:97.9; H:2.1 |  |
| **W** | 100 | W:100 |  |
| **E** | 91.1 | E:91.1; K:8.9 |  |
| **N** | 89.5 | N:89.5; Q:8.3; E:2.2 |  |
| **Q** | 99 | Q:99; K:0.5; L:0.4; E:0.1 |  |
| **-** | 98.4 | -:98.4; K:1.6 |  |
| **K** | 77.5 | K:77.5; E:22.5 |  |
| **SSLENYIK** (ID1) |  |  | 72.95 |
| **S** | 51.4 | S:51.4; K:39.1; L:9.4; -:0.1 | |
| **S** | 99.9 | S:99.9; -:0.1 |  |
| **L** | 81.4 | L:81.4; A:18.5; -:0.1 |  |
| **D** | 49.7 | D:49.7; E:31.7; N:18.5; -:0.1 | |
| **D** | 49.7 | D:49.7; N:31.7; S:18.5; -:0.1 | |
| **Y** | 99.9 | Y:99.9; -:0.1 |  |
| **I** | 87.7 | I:87.7; V:12.2; -:0.1 |  |
| **K** | 99.9 | K:99.9; -:0.1 |  |
| **LGVRENDK** (ID1) |  |  | 71.66 |
| **L** | 81 | L:81; F:19 |  |
| **G** | 81 | G:81; S:19 |  |
| **V** | 52.7 | V:52.7; I:28.3; G:19 |  |
| **R** | 50.2 | R:50.2; N:47.3; H:2.5 |  |
| **E** | 57.3 | E:57.3; N:28.3; K:14.4 |  |
| **N** | 89.1 | N:89.1; K:10.9 |  |
| **D** | 81 | D:81; N:19 |  |
| **K** | 81 | K:81; M:19 |  |
| **FLQEWVEHF** (DBL2) |  |  | 99.4 |
| **F** | 100 | F:100 |  |
| **L** | 100 | L:100 |  |
| **Q** | 100 | Q:100 |  |
| **E** | 100 | E:100 |  |
| **W** | 99.9 | W:99.9; L:0.1 |  |
| **V** | 100 | V:100 |  |
| **E** | 100 | E:100 |  |
| **H** | 94.7 | H:94.7; N:5.3 |  |
| **F** | 100 | F:100 |  |
| **ENESTNNK** (ID2b) |  |  | 72.44 |
| **E** | 99.9 | E:99.9; -:0.1 |  |
| **N** | 85.5 | N:85.5; I:13.7; S:0.6; -:0.1 | |
| **E** | 45.4 | E:45.4; A:21.9; K:18.9; Q:13.7; -:0.1 | |
| **S** | 85.5 | S:85.5; N:13.7; G:0.6; -:0.1 | |
| **T** | 80.1 | T:80.1; K:19.7; -:0.1; I:0.1 | |
| **D** | 67.9 | D:67.9; N:32.1 |  |
| **-** | 99.7 | -:99.7; T:0.3 |  |
| **-** | 99.7 | -:99.7; N:0.3 |  |
| **T** | 72.7 | T:72.7; N:26.4; I:0.9 |  |
| **K** | 70.1 | K:70.1; N:29; T:0.9 |  |
| **NMILGTSVNIY** (DBL3) |  |  | 96.37 |
| **N** | 100 | N:100 |  |
| **M** | 100 | M:100 |  |
| **I** | 100 | I:100 |  |
| **L** | 100 | L:100 |  |
| **G** | 100 | G:100 |  |
| **T** | 99.9 | T:99.9; S:0.1 |  |
| **S** | 99.9 | S:99.9; N:0.1 |  |
| **V** | 100 | V:100 |  |
| **N** | 92.9 | N:92.9; S:7.1 |  |
| **I** | 67.4 | I:67.4; T:32.6 |  |
| **Y** | 100 | Y:100 |  |
